# Barriers to Access to Care Evaluation Scale - Proxy Report (BACE-PR): Evidence of Reliability and Validity for Caregivers Reporting on Children and Adolescents with Mental Health Concerns in Greece

**DOI:** 10.1007/s10488-025-01466-7

**Published:** 2025-08-25

**Authors:** Konstantinos Kotsis, Graham Thornicroft, Julia Luiza Schafer, Aspasia Serdari, Maria Basta, Caio Borba Casella, Lauro Estivalete Marchionatti, Mauricio Scopel Hoffmann, Alexandra Tzotzi, Andromachi Mitropoulou, André Simioni, Katerina Papanikolaou, Anastasia Koumoula, Giovanni Abrahão Salum

**Affiliations:** 1 Child and Adolescent Mental Health Initiative (CAMHI), Stavros Niarchos Foundation & Child Mind Institute, Athens, Greece; 2 Child Mind Institute, 215 E 50th St, New York, NY 10022 USA; 3 Department of Psychiatry, Community CAMHS, Faculty of Medicine, School of Health Sciences, University of Ioannina, Ioannina, Greece; 4 Health Service and Population Research, Institute of Psychiatry, Psychology and Neuroscience, King’s College London, London, UK; 5 Department of Child and Adolescent Psychiatry, Medical School, Democritus University of Thrace, Alexandroupolis, Greece; 6 Department of Psychiatry, University of Crete, Heraklion, Greece; 7 Department of Child and Adolescent Psychiatry, University of Crete, Heraklion, Greece; 8 Department of Psychiatry, Universidade do Estado de São Paulo (USP), São Paulo, Brazil; 9 Department of Psychiatry, Universidade Federal do Rio Grande do Sul (UFRGS), Porto Alegre, Brazil; 10 Department of Neuropsychiatry, Universidade Federal de Santa Maria (UFSM), Santa Maria, Brazil; 11 Care Policy and Evaluation Centre, London School of Economics and Political Science, London, UK; 12 National Center for Innovation and Research in Mental Health (CISM), São Paulo, Brazil; 13 Department of Child and Adolescent Psychiatry, Agia Sophia Children’s Hospital, National and Kapodistrian University of Athens, Athens, Greece

**Keywords:** Access, Barriers to care, Caregivers, Children, Mental health care seeking, Psychometric

## Abstract

To improve access to mental health care for children and adolescents, it is necessary to identify the barriers faced by their caregivers. The aim of this study is to identify these barriers in Greece and to investigate the reliability and validity of the modified version of the Barriers to Access to Care Evaluation scale (BACE) - the BACE Proxy Report (BACE-PR). A total of 265 caregivers who reported that their offspring had mental health difficulties completed the BACE-PR. Descriptive statistics were used to identify the major barriers to accessing care. Exploratory Factor Analysis (EFA) and Confirmatory Factor Analysis (CFA) were used to investigate the factor structure of the instrument. Item parameters were assessed via Item Response Theory. Interpretability was assessed by linking summed scores to IRT-based scores. Caregivers reported care costs, their willingness to resolve problems on their own, and their own concern that their children might be seen as weak, as the major barriers to services access. Obsessive compulsive symptoms and self-harm were the conditions for which caregivers reported the highest level of barriers. EFA and CFA suggested that a one-factor solution fit the data well (RMSEA = 0.048, CFI = 0.991, TLI = 0.990). Internal consistency was found to be high (ω = 0.96). Average z-scores provided five meaningful levels of caregivers’ perceived barriers compared to the national average. Caregivers face a variety of barriers to access mental health care for their children, and this could partly explain the treatment gap in the Greek mental health sector. Our study provides evidence for the reliability and validity of the BACE-PR scale, which can aid to identify caregiver-perceived barriers and to design interventions to improve access to mental health care.

**Supplementary Information:**

The online version contains supplementary material available at 10.1007/s10488-025-01466-7.

## Introduction

Nearly half of mental disorders emerge before the age of 18 (Solmi et al., 2022). It is well established that mental disorders are leading causes of disability and, if left untreated, may have a serious impact on educational achievement and social functioning (Arias et al., 2022; Egan et al., 2016; Schlack et al., 2021)., Studies from all over the world, suggest that a large proportion of children with treatable mental health disorders do not receive the treatment they need to get better (Hall, S., Fildes, J., Perrens, B., Plummer, J., Carlisle, E., Cockayne, N., and Werner-Seidler, A. 2019; Merikangas et al., 2011; Raven et al., 2017; Whitney & Peterson, 2019). This treatment gap highlights the significant barriers to accessing care within each country’s mental health system. To increase access to mental health care services for children and adolescents, it is crucial to thoroughly understand the factors that facilitate or inhibit their ability to receive care and to have reliable instruments that can assess those barriers accurately from parents’ perspectives.

In child and adolescent mental health, caregivers play a major role in facilitating help-seeking and often serve as the primary point of contact with mental health services (Hansen et al., 2021; Yurgelun-Todd, 2023). For in-person help seeking services, research has found that family is the dominant influence for adolescents (Rickwood et al., 2015). Caregivers, as key facilitators of their children’s entry into mental health care, may encounter various barriers, including difficulties in recognizing symptoms, attitudes and stigma toward mental health that influence decisions to seek treatment, negative perception of mental health services, transport issues, flexibility of appointments and lack of knowledge about the appropriate type of care to seek (Collins et al., 2004; Drent et al., 2022; Logan & King, 2002; Owens et al., 2002; Teagle, 2002; Thornicroft et al., 2022; Yurgelun-Todd, 2023).

The literature on barriers to accessing either use an open-ended approach, asking caregivers to describe their reasons for not seeking help or the challenges they faced in accessing mental health services for their children (Eapen and Ghubash 2004; Shun Wilson Cheng et al. 2013) or employs predefined lists of barriers where caregivers indicate the presence or absence of specific barriers or rated them on Likert scale. Examples of such instruments include the Obstacle to Engagement Scale (Dumas et al. 2007), the barriers to participation scale (Kazdin et al. 1997) and Barriers to Treatment Utilization (Thurston and Phares 2008).

The literature on barriers to accessing mental health care from adults’ perspective is limited in at least two important ways. First, quantitative studies -as noted above- typically using lists of barriers for caregivers to indicate their presence or absence or to rate them on a Likert scale, usually lack validation (Berger-Jenkins et al. 2012; Girio-Herrera et al. 2013; Larson et al. 2013; Shivram et al. 2009; Shun Wilson Cheng et al. 2013; Xiong et al. 2022). Second, while tools for measuring self-reported barriers in the adult population have validation studies, they are usually limited to specific contexts or populations (Pepin et al. 2015; Topkaya et al. 2016).

One of the tools that have the potential to overcome some of these challenges is the Barriers to Access to Care Evaluation (BACE v3) (Clement et al., 2012). The instrument may be used to identify key barriers to care experienced by individuals who currently use or have recently used secondary mental health services, and it shows potential utility for use with general population samples (Clement et al., 2012). Moreover, it assesses changes in barriers following intervention programs. The BACE has been developed as a self-report measure for adults experiencing mental health conditions and has been used in different countries (Alenezi et al., 2021; Fekih-Romdhane et al., 2024; Hongo et al., 2021).A multi-level conceptualization of barriers to accessing mental health care is supported by the Barriers to Access to Care Evaluation– Parent Report (BACE-PR), which captures a diverse range of barriers, that may prevent caregivers from seeking support for their children. The BACE-PR allows for the identification of barriers across individual, social, and systemic levels. At the individual level, it captures concerns such as internalized stigma, negative beliefs about mental health care, or fears related to being judged as a parent. At the social level, it includes challenges stemming from perceived discrimination, school-related stigma, or fear of negative consequences in one’s community or workplace. At the systemic level, the measure reflects structural and service-related obstacles, including difficulties accessing care, uncertainty about where to seek help, financial costs, lack of culturally appropriate services, and dissatisfaction with previous care experiences. By comprehensively addressing the diverse spectrum of potential barriers, the BACE-PR offers a nuanced perspective on the multifaceted factors—both individual and contextual—that can impede timely access to mental health services for children and adolescents.

Since BACE has been developed for adult population, validation for accessing barriers to care in the children and adolescents population is warranted.

Given this gap, as well as the fact that barriers may vary across countries due to cultural differences as well as differences in mental health system structure and facilities, the aim of the current study is twofold. First, we aim to investigate the children/adolescents caregivers’ main perceived barriers to access to mental health care in Greece. Our second aim is to test the psychometric properties of the adapted version of BACE - PR scale as a proxy measure exploring its validity to be used by caregivers of children in need of mental health care.

## Methods

### Participants

We used data from a 2022/2023 cross-sectional survey from the Child and Adolescent Mental Health Initiative (CAMHI) on the current state and needs for child and adolescent mental health in Greece based on multiple viewpoints (Koumoula et al., 2024). A nationwide sample of 1,756 caregivers participated in the online survey, answering questions related to service use and access, literacy and stigma, parenting practices, and mental health needs of their children/adolescents. Out of them, 265 caregivers answered affirmatively to the question “*Does this child/adolescent have any mental health difficulty (any psychological problem*,* any problem with his behavior or learning) that you are aware of?*”. Subsequently they completed the BACE-PR scale. Recruitment occurred through an online respondent panel provided by the research company IQVIA OneKey. This panel was developed based on census quotas, reaching participants online via social media and website campaigns, search engine optimization, panelists’ friends referrals, and affiliate networks (“Kantar Profiles Audience Network” n.d.). To avoid self-selection, the online surveys were automatically routed to respondents based on a specific algorithm. Data was collected and preserved according to the General Data Protection Regulation (GDPR) National Policy (European Parliament and The Council, 2016). Ethical approval was granted by the Research Ethics Committee of the Democritus University of Thrace [approval number: ∆ΠΘ/ΕΗ∆Ε/42772/307].

### Instrument

The Barriers to Access to Care Evaluation (BACE) scale was developed in the Health Services and Population Research Department of the Institute of Psychiatry, Psychology and Neuroscience, King’s College, England (Clement et al., 2012). The BACE scale was originally developed as a 30-item self-report instrument conceived to evaluate barriers to access to mental health. Originally, authors suggest two subscales, the stigma subscale consisting of 12 items and the non-stigma consisting of the 18 remaining items. In the current study we used a modified version, adapted for assessing barriers to care for children and adolescents as reported by caregivers. The King’s College London original lead author granted special consent for the adaptation.

#### BACE Adaptation to be a Proxy Measure in Greece

BACE was culturally adapted and translated in Greek, following reported detailed guidelines of a five-stage cultural adaptation process. Five stages included forward translation, synthesis of versions, back translation, expert committee review and pilot testing with population. The questions were modified at a pre-adaptation stage, to focus on children, e.g. “*Being unsure where to go to get professional care*” was modified to “*Being unsure where to go to get professional care for my child/adolescent*”. Work-related questions were modified to reflect child/adolescent contexts e.g. “*Concern about what people at work might think*,* say or do*” was modified to “*Concern about what people at the school of my child/adolescent might think*,* say*,* or do*”. In detail the five stages process included the following: Stage 1 involved a Greek forward translation conducted independently by two bilingual Greek native speakers with different backgrounds. The first translator was a child and adolescent psychiatrist with expertise in the concepts examined in the questionnaire, while the second translator, unfamiliar with mental health concepts, was a certified translator from an official Greek translation company. Stage 2 involved synthesizing the two Greek translations. The two forward translators collaborated to resolve discrepancies and generate a unified translation. At Stage 3, validity checking was performed through independent back translation by two bilingual English native speakers with no medical or psychological training and they were unaware of the concepts being explored. One was an administrative member of our team, while the other was a certified translator based in the USA. At Stage 4, an expert committee reviewed the original questionnaire, all forward and back translations, and relevant documentation, reaching a consensus. The committee consisted of four Greek mental health experts, including child and adolescent psychiatrists and psychologists. Their decisions aimed to achieve semantic, idiomatic, experiential, and conceptual equivalence between the original and translated versions (Beaton et al., 2000). Stage 5 involved pilot testing, where the preliminary Greek version of the instrument was pre-tested with community samples to assess the clarity of each question and response item. Participant responses were reviewed by the expert team, and items that received feedback such as “I didn’t understand anything” or “I understood a little,” or that included concerning comments from most participants, were reformulated. In the final stage, the instrument was sent to the original author for input and final approval. As for the self-report (patient adult) measure, respondents (caregivers) should indicate whether each item has ever stopped or delayed or discouraged them from getting or continuing with mental health professional care for the child/adolescent they are responsible for (modified instruction to reflect children and adolescents). Scoring includes checking one of four possible answers: not at all (0), a little (1), quite a lot (2), or a lot (3) with higher scores indicating a greater barrier. For each barrier, according to authors, three different scores may be given; (a) the mean of the response scores, (b) the percentage reporting they have experienced the barrier to any degree (i.e. the % circling 1, 2 or 3) and (c) the percentage experiencing the barrier as a major barrier (i.e. the % circling 3).

### Statistical Analysis

For the description of the BACE items, we used the mean score for each item and percentages as suggested by authors of the original scale. To explore which mental health difficulties seem to face more barriers, we plotted a heatmap of the mental health difficulties versus each barrier (item) of the BACE scale, representing the proportion of participants with a mental health difficulty that experience each of the BACE listed barriers.

Our psychometric assessment included several steps. First, since there are no studies that adapted the BACE scale to a proxy version, we performed an Exploratory Factor Analysis (EFA) to explore its underlying structure by using the Maximum Likelihood Estimator (ML) and Geomin Oblique Rotation. The selection of factors was based on the scree plot of eigenvalues and on the factor loadings. After selection of the best model, a Confirmatory Factor Analysis (CFA) was performed to explore the fit of the model to our sample, by using the Pairwise Maximum Likelihood Estimator (PML). Model fit was evaluated with the following fit indices: the Comparative Fit Index (CFI), the Tucker-Lewis Index (TLI), the Root Mean Square Error of Approximation (RMSEA), and the Standardized Root Mean-square Residual (SRMR). A good fit is indicated by the following values: SRMR < 0.6; RMSEA < 0.06; TLI and CFI > 0.95 (Hu & Bentler, 1999). Reliability analysis was performed using Cronbach alpha and by Omega (ω) coefficient for our model. Cronbach alpha assumes equal loadings (essential tau equivalence) and a value of 0.7 is considered acceptable (“Psychometric Theory” n.d.). Omega estimates the proportion of variance in the observed total score attributable to all “modeled” sources of common variance. A value of > 0.8 is considered strong (Kalkbrenner, 2023).

For interpretability- the degree to which one can assign qualitative meaning to an instrument’s quantitative scores or change in scores (Mokkink et al., 2010)- BACE has polytomous response options and therefore the graded response model (GRM) was used to estimate item parameters. We also conducted unidimensional item response theory assessments to determine where BACE provides information according to the latent trait. Moreover, we estimated the IRT factor scores of the latent variable to rank them into percentiles aiming to provide a meaningful scoring to stakeholders and researchers.

Analysis was performed using the software RStudio version 2023.12.1 (Mokkink et al., 2010; Posit team, 2024) and the packages *lavaan* (Rosseel, 2012), *psych* (Revelle, 2024), *ltm* (Rizopoulos, 2007), *and semTools* (Terrence D. Jorgensen and Sunthud Pornprasertmanit and Alexander M. Schoemann and Yves Rosseel 2022). Database sheets and the code is openly available at the following repository (https://osf.io/crz6h/).

## Results

### Participants

Sample characteristics are shown in Table 1. The majority of the respondents were female (59.6%) and were in a relationship (83.0%). Nearly all participants (98.1%) have finished the mandatory (9 years) education in Greece. The majority of caregivers reported that their child had learning difficulties (*N* = 95), attention-deficit/hyperactivity disorder (*N* = 86) and anxiety (*N* = 77).

**Table 1:** Caregiver characteristic and reported child diagnosis

	Mean	SD
*Caregivers’ age*	41.78	7.87
Child’s age	11.69	4.07
	**n**	**%**
Caregivers’ Gender (Female)	158	59.6
*Relationship status*		
Single	1	3.40
Relationship/Cohabitation/Married	220	83.0
Separated/Divorced/Widowed	36	13.6
*Educational Level*		
Mandatory (9 years of education, ISCED 1 and 2)	4	1.51
Non Mandatory (3 years of education, ISCED 3)	98	37.0
Higher (Bachelor, Master or PhD, ISCED 6 or 7 or 8)	162	61.1
Other	1	0.37
*Income*		
Less than 1000€ monthly	89	33.6
Between 1001 to 2,000€ monthly	107	40.4
Above 2000€ monthly	58	21.9
I don’t know/Not applicable	11	4.1
*Child reported mental health difficulties by caregivers*		
Autism	25	9.4
Intellectual difficulties	13	4.91
Learning difficulties	95	35.8
Attention-deficit Hyperactivity Disorder (ADHD)	86	32.5
Excessive worries, or fears (anxiety)	77	29.1
Sadness, loss of pleasure and/or irritability (depression)	26	9.8
Headstrong and oppositional behaviors (conduct difficulties)	32	12.1
Obsessions and/or compulsions (Obsessive Compulsive Disorder)	13	4.9
Eating and weight problems	28	10.6
Delusions and hallucinations	2	0.7
Alcohol and drugs	6	2.2
Sleep difficulties	38	14.3
Self-harm and/or suicidal ideation/attempts	5	1.8
Nighttime enuresis (Bed wetting)	13	4.9
Other	14	5.2

*Total percentages of mental health difficulties exceed 100% since the item was a tickbox guiding caregivers to mark all that apply; ISCED = International Standard Classification of Education

### Barriers to Access to Care: Descriptive Assessment

**Fig. 1:**
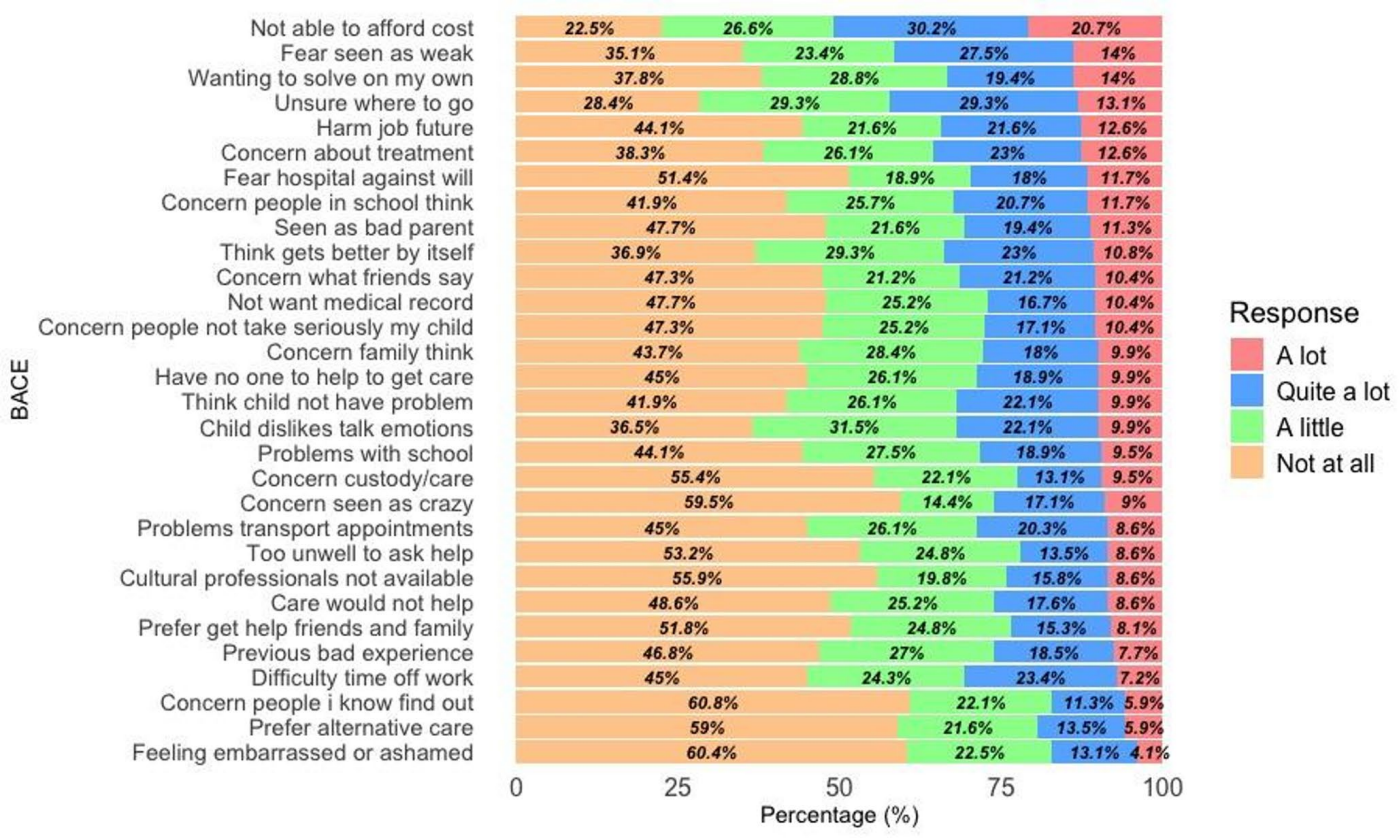
Caregivers ratings in each response (in percentage) of how much (“a lot”, “quite a lot”, “a little”, “not at all”) each item ever stopped, delayed or discouraged them from getting, or continuing with, professional care for a mental health problem. Descending order based on the percentage reported as a major barrier (circling “A lot”)

Mean scores of each item of BACE are presented in Table 2, while percentages reported each item as a barrier to any degree and as a major barrier are presented in Fig. 1. All scores lie between 0.61 and 1.49 indicating that caregivers face overall barriers to a small degree. The barrier with the highest mean score is “*Not being able to afford the financial costs involved*” which concurrently is also the item with the highest percentage reporting this as a barrier to any degree as well as reporting it as a major barrier. Other highly reported barriers include the fear that the child might be seen as weak, the willingness of the caregivers to solve the difficulties by themselves, the uncertainty of where they should ask for help and the future consequences relating to job applications.

**Table 2:** Mean scores, frequencies and ranks for each barrier in the barriers to access to care evaluation scale

No	Barrier	Mean	SD	% reporting barrier to any degree	% reporting as major barrier (‘a lot’)	Rank (1 = item has highest proportion rating as a major barrier)
11	Not being able to afford the financial costs involved.	1.49	1.06	77.48	20.72	1
2	Wanting to solve the problem of my child/adolescent on my own.	1.09	1.06	62.16	13.96	2
3	Concern that my child/adolescent might be seen as weak for having a mental health problem.	1.2	1.07	64.86	13.96	3
1	Being unsure where to go to get professional care for my child/adolescent	1.27	1.02	71.62	13.06	4
5	Concern that it might harm the child/adolescent’s chances when applying for jobs in the future	1.03	1.08	55.86	12.61	5
20	Concerns about treatments available for my child/adolescent (e.g., medication side effects).	1.1	1.05	61.71	12.61	5
4	Fear of my child/adolescent being put in hospital against his/her will.	0.9	1.08	48.65	11.71	7
28	Concern about what people at the school of my child/adolescent might think, say, or do.	1.02	1.05	58.11	11.71	8
14	Concern that I might be seen as a bad parent.	0.94	1.06	52.25	11.26	9
7	Thinking the problem of my child/adolescent would get better by itself.	1.08	1.02	63.06	10.81	10
19	Concern that people might not take my child/adolescent seriously if they found out he/she was having professional care.	0.91	1.03	52.7	10.36	11
21	Not wanting a mental health problem to be on the medical records of my child/adolescent.	0.9	1.03	52.25	10.36	12
26	Concern about what the friends of my child/adolescent might think, say, or do.	0.95	1.05	52.7	10.36	13
8	Concern about what my child/adolescent’s family might think, say, do or feel.	0.94	1.01	56.31	9.91	14
18	My child/adolescent dislikes talking about feelings, emotions, or thoughts.	1.05	0.99	63.51	9.91	15
25	Thinking my child/adolescent did not have a problem.	1	1.02	58.11	9.91	16
30	Having no one who could help me get professional care for my child/adolescent	0.94	1.02	54.95	9.91	17
24	Concern that the adolescent’s children may be taken into care or that he/she may lose access or custody without his/her agreement.	0.77	1.01	44.59	9.46	18
29	Having problems with school while my child/adolescent receives professional care.	0.94	1	55.86	9.46	19
12	Concern that my child/adolescent might be seen as ‘crazy’.	0.76	1.04	40.54	9.01	20
6	Problems with transport or traveling appointments.	0.92	1	54.95	8.56	21
13	Thinking that professional care for my child/adolescent would not help.	0.86	0.99	51.35	8.56	22
15	Professionals from the child/adolescent’s own ethnic or cultural group not being available.	0.77	1.01	44.14	8.56	23
16	Being too unwell to ask for help for my child/adolescent.	0.77	0.98	46.85	8.56	24
23	Preferring to get help from family or friends for my child/adolescent.	0.8	0.98	48.2	8.11	25
22	Having had previous bad experiences with professional care for the mental health of my child/adolescent.	0.87	0.97	53.15	7.66	26
27	Difficulty taking time off work to take my child/adolescent to a health care service or professional.	0.93	0.99	54.95	7.21	27
10	Preferring to get alternative forms of care for my child/adolescent (e.g., traditional/religious healing or alternative/complementary therapies).	0.66	0.92	40.99	5.86	28
17	Concern that people I and my child/adolescent know might find out.	0.62	0.9	39.19	5.86	29
9	Feeling embarrassed or ashamed by my child/adolescent.	0.61	0.86	39.64	4.05	30

**Fig. 2:**
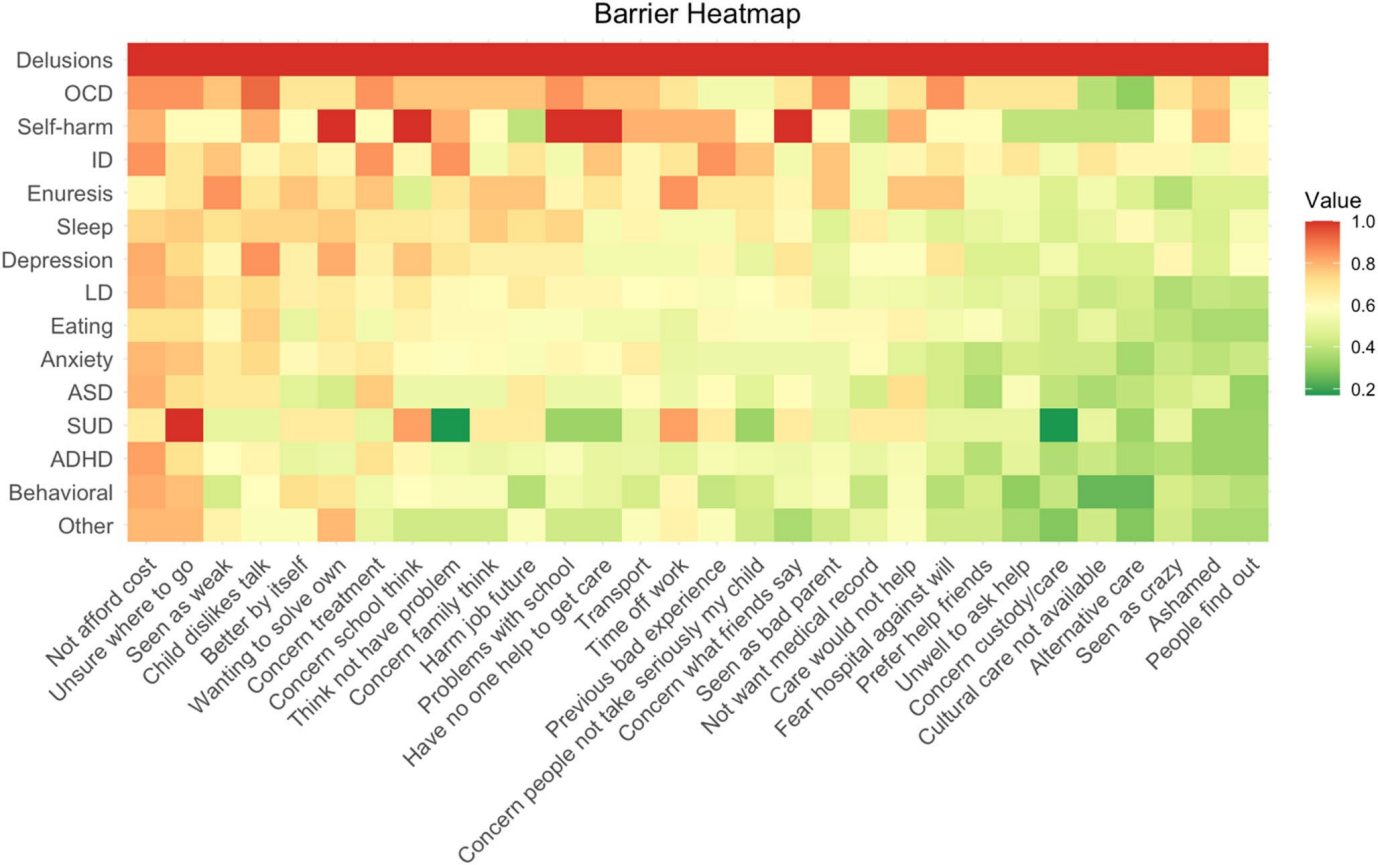
Percentage of caregivers reporting each barrier to any degree (answer: “A little”, “Quite a lot”, “A lot”) in each diagnosis. Order of BACE items according to percentage reported to any degree (descending left to right). Order of the mental health difficulties according to the overall level of barriers based on the mean BACE item scores (descending up to bottom)

Figure 2 presents the barrier heatmap according to each reported mental health difficulty. Parents reported facing all barriers in high degree when their children presented with delusions and hallucinations, while obsessive compulsive disorder was the disorder where caregivers encoutnered a lot of barriers. Other difficulties that face a lot of barriers in high degree include self-harm and intellectual disability. On the other hand, difficulties with a low level of barriers are mainly behavioral disorders as well as attention-deficit/hyperactivity disorder and autism spectrum disorder.

### Exploratory Factor Analysis

The results of the scree plot (Figure S1, Supplementary Material) favored a unidimensional solution. All 30 factor loadings were very high (> 0.6) except two that displayed values of 0.38 and 0.59, although still significant.

### Confirmatory Factor Analysis

The unidimensional model (barriers to access to care) showed very good fit indices to the data (RMSEA = 0.048, CFI = 0.991, TLI = 0.990, SRMR = 0.061). Factor loadings were high, ranging from 0.38 to 0.89 (Table 3).

### Reliability

Unidimensional model presented with high reliability with values of 0.96 to McDonald’s ω as well as Cronbach’s alpha, indicating excellent internal consistency (Table 3).

**Table 3:** Confirmatory factor analysis parameters and reliability coefficients

No	Item	Factor loadings	Threshold 1	Threshold 2	Threshold 3
1	Being unsure where to go to get professional care for my child/adolescent.	0.509	−0.594	0.179	1.127
2	Wanting to solve the problem of my child/adolescent on my own.	0.549	−0.341	0.384	1.099
3	Concern that my child/adolescent might be seen as weak for having a mental health problem.	0.763	−0.392	0.192	1.017
4	Fear of my child/adolescent being put in hospital against his/her will.	0.753	0.070	0.530	1.164
5	Concern that it might harm the child/adolescent’s chances when applying for jobs in the future	0.786	−0.146	0.392	1.109
6	Problems with transport or traveling appointments.	0.684	−0.127	0.556	1.314
7	Thinking the problem of my child/adolescent would get better by itself.	0.660	−0.314	0.393	1.150
8	Concern about what my child/adolescent’s family might think, say, do or feel.	0.841	−0.164	0.522	1.267
9	Feeling embarrassed or ashamed by my child/adolescent.	0.795	0.261	0.863	1.552
10	Preferring to get alternative forms of care for my child/adolescent (e.g., traditional/religious healing or alternative/complementary therapies).	0.835	0.209	0.842	1.429
11	Not being able to afford the financial costs involved.	0.375	−0.728	−0.029	0.850
12	Concern that my child/adolescent might be seen as ‘crazy’.	0.835	0.256	0.631	1.257
13	Thinking that professional care for my child/adolescent would not help.	0.743	−0.090	0.592	1.277
14	Concern that I might be seen as a bad parent.	0.757	−0.024	0.550	1.273
15	Professionals from the child/adolescent’s own ethnic or cultural group not being available.	0.720	0.127	0.654	1.333
16	Being too unwell to ask for help for my child/adolescent	0.799	0.078	0.709	1.352
17	Concern that people I and my child/adolescent know might find out.	0.894	0.256	0.867	1.404
18	My child/adolescent dislikes talking about feelings, emotions, or thoughts.	0.730	−0.384	0.444	1.247
19	Concern that people might not take my child/adolescent seriously if they found out he/she was having professional care.	0.833	−0.078	0.560	1.236
20	Concerns about treatments available for my child/adolescent (e.g., medication side effects).	0.684	−0.324	0.342	1.140
21	Not wanting a mental health problem to be on the medical records of my child/adolescent.	0.743	−0.084	0.572	1.266
22	Having had previous bad experiences with professional care for the mental health of my child/adolescent.	0.781	−0.128	0.642	1.444
23	Preferring to get help from family or friends for my child/adolescent.	0.816	0.042	0.690	1.364
24	Concern that the adolescent’s children may be taken into care or that he/she may lose access or custody without his/her agreement.	0.853	0.189	0.741	1.276
25	Thinking my child/adolescent did not have a problem.	0.733	−0.234	0.430	1.239
26	Concern about what the friends of my child/adolescent might think, say, or do.	0.819	−0.125	0.489	1.261
27	Difficulty taking time off work to take my child/adolescent to a health care service or professional.	0.654	−0.114	0.448	1.346
28	Concern about what people at the school of my child/adolescent might think, say, or do.	0.771	−0.217	0.439	1.201
29	Having problems with school while my child/adolescent receives professional care.	0.789	−0.172	0.546	1.260
30	Having no one who could help me get professional care for my child/adolescent	0.793	−0.110	0.568	1.350
	*Model Fit*				
	RMSEA	0.048			
	CFI	0.991			
	TLI	0.990			
	SRMR	0.061			
	*Reliability*				
	McDonald’s ω	0.96			
	Cronbach’s alpha	0.96			

Note: RMSEA = root-mean-square error of approximation; CFI = comparative fit index; TLI = Tucker–Lewis index; SRMR = standardized root-mean-square residual;

### Interpretability

*Unidimensional Item Performance Analysis*. Item response function curves and item information curves for each item can be found in Figures S3 and S4, supplemental material. Test information function plot (Figure S5) shows that BACE proxy version provides the most information about slightly-above-than-average barrier levels (the peak is around θ = 1).

**Fig. 3:**
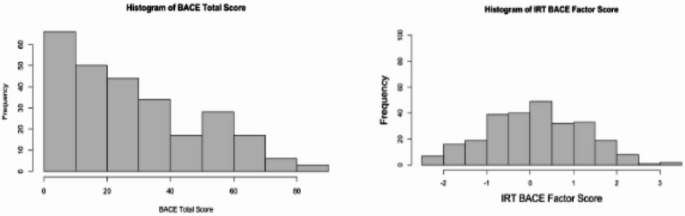
Histogram of the BACE Factor score from the Item Response Analysis

​​*Linking summed score to IRT-based z-scores*. Factor score from the IRT of the BACE proxy version as shown in Fig. 3 follows the normal distribution. The z-scores for the latent variable (Table 4) provide a reference point to assess interpretability of the BACE. Based on those scores, we classified the amount of barriers that caregivers face as: (1) No barriers (BACE total = 0); (2) slight barriers (BACE total 1–31); (3) mild barriers (BACE total 32–44); (4) moderate barriers (BACE total 45–63); and (5) severe barriers (BACE total over 64).

**Table 4:** Interpretation of the barriers to access to care evaluation scale total score

BACE total score	Avg IRT z-score	Percentile	Interpretation
0	−2.15	1.71	No barriers
1	−1.78	4.50	Slight barriers
2	−1.71	5.50	Slight barriers
3	−1.62	6.67	Slight barriers
4	−1.35	10.25	Slight barriers
5	−1.17	13.13	Slight barriers
6	−0.99	16.00	Slight barriers
7	−0.98	16.00	Slight barriers
8	−0.98	16.00	Slight barriers
9	−0.90	17.40	Slight barriers
10	−0.71	21.30	Slight barriers
11	−0.58	24.25	Slight barriers
12	−0.56	24.83	Slight barriers
13	−0.54	27.43	Slight barriers
14	−0.50	28.46	Slight barriers
15	−0.46	29.50	Slight barriers
16	−0.43	29.80	Slight barriers
17	−0.33	34.75	Slight barriers
18	−0.20	37.30	Slight barriers
19	−0.08	39.86	Slight barriers
20	−0.08	40.00	Slight barriers
21	−0.01	41.50	Slight barriers
22	0.05	43.00	Slight barriers
23	0.10	44.42	Slight barriers
24	0.14	45.83	Slight barriers
25	0.19	47.25	Slight barriers
26	0.30	50.43	Slight barriers
27	0.30	51.50	Slight barriers
28	0.30	52.07	Slight barriers
29	0.34	52.63	Slight barriers
30	0.39	53.20	Slight barriers
31	0.45	55.30	Slight barriers
32	0.51	57.40	Mild barriers
33	0.57	59.50	Mild barriers
34	0.57	59.58	Mild barriers
35	0.58	59.67	Mild barriers
36	0.59	60.46	Mild barriers
37	0.62	61.25	Mild barriers
38	0.68	63.00	Mild barriers
39	0.74	64.17	Mild barriers
40	0.75	64.44	Mild barriers
41	0.76	64.72	Mild barriers
42	0.77	65.00	Mild barriers
43	0.89	67.00	Mild barriers
44	0.99	69.50	Mild barriers
46	1.05	70.67	Moderate barriers
47	1.11	71.83	Moderate barriers
49	1.18	73.00	Moderate barriers
50	1.20	74.33	Moderate barriers
51	1.22	75.67	Moderate barriers
52	1.23	76.00	Moderate barriers
53	1.24	76.33	Moderate barriers
54	1.25	78.33	Moderate barriers
55	1.35	80.33	Moderate barriers
57	1.41	82.67	Moderate barriers
58	1.48	85.00	Moderate barriers
59	1.69	89.33	Moderate barriers
60	1.71	90.67	Moderate barriers
61	1.74	92.00	Moderate barriers
62	1.76	93.33	Moderate barriers
63	1.79	94.67	Moderate barriers
64	2.04	96.00	Severe barriers
65	2.04	96.17	Severe barriers
66	2.05	96.33	Severe barriers
67	2.05	96.50	Severe barriers
69	2.05	96.67	Severe barriers
71	2.06	96.83	Severe barriers
72	2.06	97.00	Severe barriers
73	2.09	97.20	Severe barriers
77	2.13	97.40	Severe barriers
79	2.16	97.60	Severe barriers
80	2.19	97.80	Severe barriers
81	2.22	98.00	Severe barriers
84	2.59	99.00	Severe barriers
88	2.78	99.00	Severe barriers

## Discussion

The aim of this study was to identify the barriers that caregivers face to access mental health care in Greece for their children and to explore the reliability and validity of the culturally adapted BACE-PR in a nationwide sample of caregivers with mental health concerns of their children. The top five major barriers to access care in Greece were “Not being able to afford the financial costs involved”, “Wanting to solve the problem of my child/adolescent on my own’’, “Concern that my child/adolescent might be seen as weak for having a mental health problem”, “Being unsure where to go to get professional care for my child/adolescent” and “Concern that it might harm the child/adolescent’s chances when applying for jobs in the future”. We showed consistent evidence for the reliability and validity of BACE-PR as a unidimensional construct. We also provide practical recommendations for interpretability of the BACE-PR by means of using IRT based scores (z-scores).

Several factors contribute to barriers in mental health care. It is well known that countries allocate on average only 2.1% of their total health budgets on mental health, resulting in limited resources (Knapp et al., 2006; World Health Organization, 2021). Given the shortage of mental health professionals (Saraceno et al., 2007; World Health Organization, 2021) worldwide, limited recourses become more evident, leaving population needs unmet. Additionally, these longstanding challenges have been further amplified recently by the COVID-19 pandemic which led to an increase in mental health problems and consequently, an increased demand for services (World Health Organisation, 2020).

In general, these factors also apply to Greece (a country with approximately 10 million inhabitants) which was additionally affected by the financial crisis. At a political level, public funding for mental health was reduced by 20% between 2010 and 2011, and by an additional 55% between 2011 and 2012 (Anagnostopoulos & Soumaki, 2012). At an individual level, it is not surprising that one of our study’s most significant findings is that three quarters of caregivers reported financial costs as a barrier to accessing mental health care. We argue that this may be related to the consequences of 12 years of continuous financial crisis in Greece, which appeared to worsened due to increased demands following the COVID-19 pandemic. During the crisis years, unemployment rates rose, and income loss was observed across most professions. As a result, with reduced earnings, the population faced increasing difficulty in affording healthcare expenses. Moreover, mental health is known to be particularly vulnerable to rapid economic fluctuations (Durkheim, 2006; Economou et al., 2011; United Nations Publications, 2014). Notably, the proportion of the population that is at risk of poverty or social exclusion rose from 28.1% in 2008 to 36% in 2014 and 35.7% in 2015 (Stylianidis & Souliotis, 2019). Consequently, demand for mental health services increased, while at the same time public mental health services operated with fewer employees (compared to pre-crisis years) with long waiting lists especially since the vast majority of child and adolescent psychiatrists work in the private sector (Kotsis et al., 2019; Marchionatti et al., 2024). Although Greece has one of the highest ratios of child and adolescent psychiatrists per 100,000 young people (Signorini et al., 2017), a significant shortage persists in the public sector. This issue was further exacerbated by the financial crisis due to hiring freezes. However, the lack of Child and Adolescent Mental Health Services (CAMHS), particularly outside major urban centers, has long been a persistent challenge. Although the public mental health system is mainly free of charge, many caregivers seek care in the private sector. However, the cost of private treatment for children with mental and neurodevelopmental disorders is only partially covered by the national insurance fund, thus caregivers often have to pay out-of-pocket costs for their child’s treatment. Moreover, since there are areas, especially rural ones, with total lack of child psychiatrists (even in the private sector), caregivers are struggling to find a physician to prescribe the interventions to be covered by the national insurance. Our finding is consistent with the international literature where in many studies the cost consistently emerges as a major barrier (Bussing et al., 2003; Harwood et al., 2009; Sawyer et al., 2004).

Another significant finding of our study is that a set of barriers to mental health access among caregivers of children with mental health problems in Greece are related to attitudes and stigma. Several studies in the literature suggest that these types of barriers are noted among caregivers (Cohen et al., 2012; Meredith et al., 2009; Pullmann et al., 2010). To our knowledge, no prior studies in Greece have explored caregivers’ beliefs regarding barriers to their children’s mental health services. However, stigma surrounding mental illness is prevalent in Greek culture according to a systematic review (Tzouvara et al., 2016). Finally, the lack of knowledge about where to refer for help also represents a barrier in our study, consistent with the literature (Cohen et al., 2012; Sayal et al., 2015; Stein et al., 2003). This may reflect the fragmentation of the Greek mental health system, the significant disparities in standards of care across both public and private providers and the overall challenges the system faces in Greece (Loukidou et al., 2013; Petrea et al., 2020). Current initiatives aim to mitigate those barriers by improving public access to information (www.camhi.gr).

Based on our study, in Greece caregivers of children with psychosis, obsessive compulsive symptoms (OCS), self-harm behaviors, or intellectual disability (ID) seem to face the most barriers and to a higher level compared to caregivers of children with other disorders in our sample. These conditions are severe, often associated with significant impairment in everyday functioning and may need specialized services. Patients with psychosis do not seek help and face a lot of barriers (stigma, mental health literacy) while poor service engagement is common in adolescents (Birchwood et al., 1998; Skrobinska et al., 2024). Additionally, considering that psychotic patients may require inpatient treatment, our finding is not surprising given the significant lack of inpatient units in Greece (Marchionatti et al., 2024). Parents of children with OCD find themselves caught in “loops” engaging in repeated process steps due to the emergence of barriers, when they seek exposure therapy. They also feel isolated and guilty because of the burden of finding treatment for their children (Frank et al., 2023). Moreover, poor mental health literacy and stigmatizing attitudes contribute to delayed help seeking for OCD (Chaves et al., 2022). Similar reasons seem to apply to help seeking for young people at risk of self-harm (Aggarwal et al., 2021; Waller et al., 2023). It is well known that stigma is high for youth’s self-harm behaviors and the care they receive in the emergency departments is often inadequate (Aggarwal et al., 2021; Cadorna et al., 2023; Zanus et al., 2017). Caregivers of children with ID also indicate they face high levels of barriers that might be related to the barriers they also face in educational settings. Furthermore, when ID is comorbid with other disorders, such as autism spectrum disorders (ASD), it increases the likelihood of unmet mental health care needs (Menezes et al., 2021). Moreover, in the case of Greece we should also highlight structural barriers such as the lack of specialized services providing evidence-based treatment for these conditions, especially in the public sector. On the other hand, our findings showed that behavioral disorders and other neurodevelopmental disorders such as autism spectrum disorder and attention deficit hyperactivity disorder face a lower level of barriers when compared to other mental health concerns. We hypothesize that externalizing problems as well as communication deficits and social impairment in ASD may prompt earlier help seeking, coupled with greater availability of private services in Greece that provide care for these conditions.

We also found that the psychometric testing of the BASE-PR provides good evidence for data quality and internal consistency. The unidimensional model fits the data well indicating that the BACE-PR can be used to measure the barriers that the caregivers face when they need to access mental health care for their children. The unidimensional solution seems in contrast with the literature considering that the original version of the BACE divides the items as stigma non-related and stigma (Clement et al., 2012), which was corroborated by the Arabic, the Japanese (excluding 6 items and with marginal fit indices) and the French versions (Alenezi et al., 2021; Fekih-Romdhane et al., 2024; Hongo et al., 2021). Our unique finding of unidimensionality might be explained by the fact that our tool represents a modified proxy version. Based on that, we suggest that the BACE-PR scale should be treated as a measure of the overall perceived barriers. In terms of practicality and interpretability, the results of this study can be used by stakeholders to understand the quality and quantity of the barriers caregivers face. Reliability of our proxy version was also found very high, consistent with the above-mentioned translations.

### Limitations and Strengths

Our study has important limitations. First, the sample size is small to conceptualize which barriers caregivers face in Greece and to explore measurement invariance across different groups. Future studies with larger sample sizes will also enable the identification of barriers faced by caregivers from diverse backgrounds (e.g., minorities) or other demographic groups, such as those living in rural areas or low-income families, where barriers appear to be distinct (Crumb et al., 2019). The sample size also prevented us from conducting EFA and CFA in distinct samples, as recommended by the literature for psychometric assessment of psychological instruments (Lorenzo-Seva, 2022; Schmitt et al., 2018). Second, this is an online panel-based sample, thus implying representative bias for groups without internet access. Moreover, a key limitation of this study is its cross-sectional design, which precludes the assessment of test-retest reliability. Finally, a limitation of the translation procedure is the lack of an assessment of experts’ agreement on the content validity of each item. Our study also has strengths that should be highlighted. To the best of our knowledge this is the first study using a modified version of BACE, providing the international community with a tool to assess barriers to accessing care in children and adolescents. In addition, it is the first study in Greece exploring the barriers that caregivers face in accessing to mental health care. Second, we followed a rigorous cross-adaptation process for the instrument. Third, item performance analyses in a nationwide survey, provided psychometric evidence of BACE-PR adequacy on an item-based approach, surpassing the limitations of classical test theory analyses. Finally, our study provides Greek service providers and stakeholders with a valid tool to record caregivers’ perceived barriers to design interventions to address them.

## Conclusions

In this study, we developed and validated the BACE-PR tool, addressing a gap of tools for caregiver report of barriers to child and adolescent mental health care. Caregivers of children with mental health concerns may encounter numerous barriers when help from mental health professionals is needed. Identifying them is important to understand the treatment gap at both the national and at the local level considering the different needs across the country, especially in terms of the uneven distribution of services and professionals. The network of interrelated barriers we identified, emphasizes the imperative for a coordinated, multi-tiered intervention strategy. Financial difficulties emerged as a key obstacle, reinforcing the urgency of providing free-of-charge, specialized services to ensure that economic hardship does not prevent families from accessing care. In parallel, stigma and limited awareness about mental health remain significant deterrents to help-seeking, highlighting the importance of public education and anti-stigma initiatives aimed at caregivers and communities. Beyond individual and social factors, systemic challenges such as lack of information about available services further compound difficulties in accessing care. Addressing these barriers requires practical solutions such as service mapping, clear referral pathways, and improved accessibility of culturally responsive services. Taken together, the findings point to the need for comprehensive strategies that engage all levels of the care system—from individual and family-level interventions to community and structural reforms. Such an approach is essential to promote timely, equitable, and effective mental health support for all children and adolescents, particularly those from underserved or vulnerable populations.

To improve access to treatment and to provide early and comprehensive services, we also need to explore variations in the perceived barriers among caregivers of children across different mental health disorders and different ages. Finally, the present study supports the use of BACE-PR to identify perceived barriers. Future studies on larger scale populations based on this tool are needed to confirm our findings. Furthermore, appropriate interventions may be applied by policy makers to address the barriers, and longitudinal studies may evaluate their effect on children’s mental health in Greece. Moreover, specifically addressing barriers at a more regional level can also be a positive strategy for fighting barriers considering the different needs across the country, especially in terms of the uneven distribution (against rural areas) of services and professionals. Diminishing the barriers and therefore increasing access to mental health care would be extremely beneficial for children and adolescents, since it will allow for early intervention and overall decrease the treatment gap.

## Supplementary Information

Below is the link to the electronic supplementary material.

Supplementary Material 1

## Data Availability

The dataset is openly available in the CAMHI Open Science Framework repository [10.17605/OSF.IO/CRZ6H*]*.

## Code Availability

The statistical codes supporting the conclusions of the current study are openly available in the CAMHI Open Science Framework repository [10.17605/OSF.IO/CRZ6H*]*.

## References

[CR1] Aggarwal, S., Borschmann, R., & Patton, G. C. (2021). Tackling stigma in self-harm and suicide in the young. *The Lancet Public Health*, *6*(1), e6–e7. 10.1016/S2468-2667(20)30259-0 33417848 10.1016/S2468-2667(20)30259-0 PMC7611270

[CR2] Alenezi, A. F., Aljowder, A., Almarzooqi, M. J., Alsayed, M., Aldoseri, R., Alhaj, O., et al. (2021). Translation and validation of the Arabic version of the barrier to access to care evaluation (BACE) scale. *Mental Health and Social Inclusion*, *25*(4), 352–365. 10.1108/mhsi-05-2021-0022

[CR3] Anagnostopoulos, D. K., & Soumaki, E. (2012). The impact of socio-economic crisis on mental health of children and adolescents. *Psychiatrike*, *23*(1), 13–16. https://www.ncbi.nlm.nih.gov/pubmed/22549036 22549036

[CR4] Arias, D., Saxena, S., & Verguet, S. (2022). Quantifying the global burden of mental disorders and their economic value. *EClinicalMedicine*, *54*, 101675. 10.1016/j.eclinm.2022.101675 36193171 10.1016/j.eclinm.2022.101675 PMC9526145

[CR5] Beaton, D. E., Bombardier, C., Guillemin, F., & Ferraz, M. B. (2000). Guidelines for the process of cross-cultural adaptation of self-report measures. *Spine*, *25*(24), 3186–3191. 10.1097/00007632-200012150-00014 11124735 10.1097/00007632-200012150-00014

[CR6] Berger-Jenkins, E., McKay, M., Newcorn, J., Bannon, W., & Laraque, D. (2012). Parent medication concerns predict underutilization of mental health services for minority children with ADHD. *Clinical Pediatrics*, *51*(1), 65–76. 10.1177/0009922811417286 21868596 10.1177/0009922811417286

[CR7] Birchwood, M., Todd, P., & Jackson, C. (1998). Early intervention in psychosis. *The British Journal of Psychiatry,**172*(S33), 53–59. 10.1192/s0007125000297663 9764127

[CR8] Bussing, R., Zima, B. T., Gary, F. A., & Garvan, C. W. (2003). Barriers to detection, help-seeking, and service use for children with ADHD symptoms. *The Journal of Behavioral Health Services & Research,**30*(2), 176–189. 10.1007/bf02289806 12710371 10.1007/BF02289806

[CR9] Cadorna, G., Vera San Juan, N., Staples, H., Johnson, S., & Appleton, R. (2023). Review: Systematic review and metasynthesis of qualitative literature on young people’s experiences of going to a&e/emergency departments for mental health support. *Child and Adolescent Mental Health*. 10.1111/camh.12683 37828704 10.1111/camh.12683

[CR10] Chaves, A., Arnáez, S., Castilla, D., Roncero, M., & García-Soriano, G. (2022). Enhancing mental health literacy in obsessive-compulsive disorder and reducing stigma via smartphone: A randomized controlled trial protocol. *Internet Interventions*, *29*(100560), 100560. 10.1016/j.invent.2022.100560 35874968 10.1016/j.invent.2022.100560 PMC9305319

[CR11] Cheng, S. W., Fenn, W., D., & Le Couteur, A. (2013). Understanding the mental health needs of Chinese children living in the North East of England. *Ethnicity and Inequalities in Health and Social Care*, *6*(1), 16–22. 10.1108/eihsc-04-2013-0005

[CR12] Clement, S., Brohan, E., Jeffery, D., Henderson, C., Hatch, S. L., & Thornicroft, G. (2012). Development and psychometric properties the barriers to access to care evaluation scale (BACE) related to people with mental ill health. *BMC Psychiatry*, *12*, 36. 10.1186/1471-244X-12-36 22546012 10.1186/1471-244X-12-36 PMC3379935

[CR13] Cohen, E., Calderon, E., Salinas, G., SenGupta, S., & Reiter, M. (2012). Parents’ perspectives on access to child and adolescent mental health services. *Social Work in Mental Health*, *10*(4), 294–310. 10.1080/15332985.2012.672318

[CR14] Collins, K. A., Westra, H. A., Dozois, D. J. A., & Burns, D. D. (2004). Gaps in accessing treatment for anxiety and depression: Challenges for the delivery of care. *Clinical Psychology Review*, *24*(5), 583–616. 10.1016/j.cpr.2004.06.001 15325746 10.1016/j.cpr.2004.06.001

[CR15] Crumb, L., Mingo, T. M., & Crowe, A. (2019). Get over it and move on: The impact of mental illness stigma in rural, low-income United States populations. *Mental Health & Prevention,**13*, 143–148. 10.1016/j.mhp.2019.01.010

[CR16] Drent, H. M., van den Hoofdakker, B., Buitelaar, J. K., Hoekstra, P. J., & Dietrich, A. (2022). Factors related to perceived stigma in parents of children and adolescents in outpatient mental healthcare. *International Journal of Environmental Research and Public Health*. 10.3390/ijerph191912767 36232067 10.3390/ijerph191912767 PMC9566109

[CR17] Dumas, J. E., Nissley-Tsiopinis, J., & Moreland, A. D. (2007). From intent to enrollment, attendance, and participation in preventive parenting groups. *Journal of Child and Family Studies*, *16*(1), 1–26. 10.1007/s10826-006-9042-0

[CR18] Durkheim, E. (2006). *On Suicide*. Penguin UK. https://play.google.com/store/books/details?id=Dk31PO6cLW4C

[CR19] Eapen, V., & Ghubash, R. (2004). Help-seeking for mental health problems of children: Preferences and attitudes in the United Arab Emirates. *Psychological Reports,**94*(2), 663–667. 10.2466/pr0.94.2.663-667 15154199 10.2466/pr0.94.2.663-667

[CR20] Economou, M., Madianos, M., Theleritis, C., Peppou, L. E., & Stefanis, C. N. (2011). Increased suicidality amid economic crisis in Greece. *The Lancet*. 10.1016/S0140-6736(11)61638-3 10.1016/S0140-6736(11)61638-3 22018009

[CR21] Egan, M., Daly, M., & Delaney, L. (2016). Adolescent psychological distress, unemployment, and the great recession: Evidence from the National Longitudinal Study of Youth 1997. *Social Science & Medicine,**156*, 98–105. 10.1016/j.socscimed.2016.03.013 27019144 10.1016/j.socscimed.2016.03.013

[CR22] European Parliament and The Council (2016). *Regulation (EU) 2016/679 of the European Parliament and of the Council. 679*:.

[CR23] Fekih-Romdhane, F., Boukadida, Y., Abassi, B., Chaibi, L. S., Conus, P., Krebs, M. O., et al. (2024). French validation of the barriers to access to care evaluation (BACE-3) scale. *L’Encephale*. 10.1016/j.encep.2023.11.020 38311478 10.1016/j.encep.2023.11.020

[CR24] Frank, H. E., Cain, G., Freeman, J., Benito, K. G., O’Connor, E., Kemp, J., & Kim, B. (2023). Parent-identified barriers to accessing exposure therapy: A qualitative study using process mapping. *Frontiers in Psychiatry,**14*, Article 1068255. 10.3389/fpsyt.2023.1068255 37020732 10.3389/fpsyt.2023.1068255 PMC10067909

[CR25] Girio-Herrera, E., Owens, J. S., & Langberg, J. M. (2013). Perceived barriers to help-seeking among parents of at-risk kindergarteners in rural communities. *Journal of Clinical Child and Adolescent Psychology: The Official Journal for the Society of Clinical Child and Adolescent Psychology American Psychological Association Division*, *53*(1), 68–77. 10.1080/15374416.2012.715365. *42*. 10.1080/15374416.2012.715365 22963042

[CR26] Hall, S., Fildes, J., Perrens, B., Plummer, J., Carlisle, E., Cockayne, N., & Werner-Seidler, A. (2019). *Can we talk? Seven year youth mental health report– 2012–2018*. Mission Australia: Sydney, NSW.

[CR27] Hansen, A. S., Telléus, G. K., Mohr-Jensen, C., & Lauritsen, M. B. (2021). Parent-perceived barriers to accessing services for their child’s mental health problems. *Child and Adolescent Psychiatry and Mental Health*, *15*(1), 4. 10.1186/s13034-021-00357-7 33514400 10.1186/s13034-021-00357-7 PMC7847149

[CR28] Harwood, M. D., O’Brien, K. A., Carter, C. G., & Eyberg, S. M. (2009). Mental health services for preschool children in primary care: A survey of maternal attitudes and beliefs. *Journal of Pediatric Psychology*, *34*(7), 760–768. 10.1093/jpepsy/jsn128 19064608 10.1093/jpepsy/jsn128 PMC2735061

[CR29] Hongo, M., Oshima, F., Nishinaka, H., Seto, M., Ohtani, T., & Shimizu, E. (2021). Reliability and validity of the Japanese version of the barriers to access to care evaluation scale version 3 for people with mental disorders: An online survey study. *Frontiers in Psychology*, *12*, 760184. 10.3389/fpsyg.2021.760184 34777164 10.3389/fpsyg.2021.760184 PMC8581348

[CR30] Hu, L. T., & Bentler, P. M. (1999). Cutoff criteria for fit indexes in covariance structure analysis: Conventional criteria versus new alternatives. *Structural Equation Modeling: A Multidisciplinary Journal,**6*(1), 1–55. 10.1080/10705519909540118

[CR31] Kalkbrenner, M. T. (2023). Alpha, omega, and *H* internal consistency reliability estimates: Reviewing these options and when to use them. *Counseling Outcome Research and Evaluation*, *14*(1), 77–88. 10.1080/21501378.2021.1940118

[CR32] Kantar Profiles Audience Network (n.d.). https://www.kantar.com/expertise/research-services/panels-and-audiences/kantar-profiles-network. Accessed 16 June 2024.

[CR33] Kazdin, A. E., Holland, L., Crowley, M., & Breton, S. (1997). Barriers to treatment participation scale: Evaluation and validation in the context of child outpatient treatment. *Journal of Child Psychology and Psychiatry and Allied Disciplines,**38*(8), 1051–1062. 10.1111/j.1469-7610.1997.tb01621.x 9413802 10.1111/j.1469-7610.1997.tb01621.x

[CR34] Knapp, M., Funk, M., Curran, C., Prince, M., Grigg, M., & McDaid, D. (2006). Economic barriers to better mental health practice and policy. *Health Policy and Planning*, *21*(3), 157–170. 10.1093/heapol/czl003 16522714 10.1093/heapol/czl003

[CR35] Kotsis, K., Giannopoulou, I., Anagnostopoulos, D., & Soumaki, E. (2019). Communications of the European society for child and adolescent psychiatry. *European Child & Adolescent Psychiatry*, *28*(7), 1005–1010. 10.1007/s00787-019-01315-7 30972582 10.1007/s00787-019-01315-7

[CR36] Koumoula, A., Marchionatti, L. E., Karagiorga, V. E., Schafer, J. L., Simioni, A., Caye, A., et al. (2024). Understanding priorities and needs for child and adolescent mental health in Greece from multiple informants: An open resource dataset. *European Child & Adolescent Psychiatry*. 10.1007/s00787-024-02400-2 10.1007/s00787-024-02400-2 PMC11564210 38558204

[CR37] Larson, J., dosReis, S., Stewart, M., Kushner, R., Frosch, E., & Solomon, B. (2013). Barriers to mental health care for urban, lower income families referred from pediatric primary care. *Administration and Policy in Mental Health*, *40*(3), 159–167. 10.1007/s10488-011-0389-1 22113729 10.1007/s10488-011-0389-1

[CR38] Logan, D. E., & King, C. A. (2002). Parental identification of depression and mental health service use among depressed adolescents. *Journal of the American Academy of Child and Adolescent Psychiatry*, *41*(3), 296–304. 10.1097/00004583-200203000-00009 11886024 10.1097/00004583-200203000-00009

[CR39] Lorenzo-Seva, U. (2022). SOLOMON: A method for splitting A sample into equivalent subsamples in factor analysis. *Behavior Research Methods*, *54*(6), 2665–2677. 10.3758/s13428-021-01750-y 34918226 10.3758/s13428-021-01750-y PMC9729132

[CR40] Loukidou, E., Mastroyiannakis, A., Power, T., Craig, T., Thornicroft, G., & Bouras, N. (2013). Greek mental health reform: Views and perceptions of professionals and service users. *Psychiatriki,**24*(1), 37–44. 23603267

[CR41] Marchionatti, L. E., Schafer, J. L., Karagiorga, V. E., Balikou, P., Mitropoulou, A., Serdari, A., et al. (2024). The mental health care system for children and adolescents in Greece: A review and structure assessment. *Frontiers in Health Services,**4*, 1470053. 10.3389/frhs.2024.1470053 39723330 10.3389/frhs.2024.1470053 PMC11668766

[CR42] Menezes, M., Robinson, M. F., Harkins, C., Sadikova, E., & Mazurek, M. O. (2021). Unmet health care needs and health care quality in youth with autism spectrum disorder with and without intellectual disability. *Autism: The International Journal of Research and Practice,**25*(8), 2199–2208. 10.1177/13623613211014721 34030515 10.1177/13623613211014721

[CR43] Meredith, L. S., Stein, B. D., Paddock, S. M., Jaycox, L. H., Quinn, V. P., Chandra, A., & Burnam, A. (2009). Perceived barriers to treatment for adolescent depression. *Medical Care*, *47*(6), 677–685. 10.1097/MLR.0b013e318190d46b 19434001 10.1097/MLR.0b013e318190d46b

[CR44] Merikangas, K. R., He, J. P., Burstein, M., Swendsen, J., Avenevoli, S., Case, B., et al. (2011). Service utilization for lifetime mental disorders in U.S. adolescents: Results of the National comorbidity survey-adolescent supplement (NCS-A). *Journal of the American Academy of Child and Adolescent Psychiatry,**50*(1), 32–45. 10.1016/j.jaac.2010.10.006 21156268 10.1016/j.jaac.2010.10.006 PMC4408275

[CR45] Mokkink, L. B., Terwee, C. B., Patrick, D. L., Alonso, J., Stratford, P. W., Knol, D. L., et al. (2010). The COSMIN study reached international consensus on taxonomy, terminology, and definitions of measurement properties for health-related patient-reported outcomes. *Journal of Clinical Epidemiology*, *63*(7), 737–745. 10.1016/j.jclinepi.2010.02.006 20494804 10.1016/j.jclinepi.2010.02.006

[CR46] Owens, P. L., Hoagwood, K., Horwitz, S. M., Leaf, P. J., Poduska, J. M., Kellam, S. G., & Ialongo, N. S. (2002). Barriers to children’s mental health services. *Journal of the American Academy of Child and Adolescent Psychiatry,**41*(6), 731–738. 10.1097/00004583-200206000-00013 12049448 10.1097/00004583-200206000-00013

[CR47] Pepin, R., Segal, D. L., Klebe, K. J., Coolidge, F. L., Krakowiak, K. M., & Bartels, S. J. (2015). The barriers to mental health services scale revised: Psychometric analysis among older adults. *Mental Health and Prevention,**3*(4), 178–184. 10.1016/j.mhp.2015.09.001 26682131 10.1016/j.mhp.2015.09.001 PMC4677330

[CR48] Petrea, I., Tsinganos, P., Fountoulakis, K., Kalpaxi, P., Koupidis, S., & Trias, M. (2020). *Mental Health Services Delivery in Greece: A Joint Rapid Assessment and Recommendations for Reform*. Hellenic Ministry of Health and World Health Organization for Europe.

[CR49] *RStudio: Integrated Development Environment for R, Posit Software, PBC*. Posit team, & Boston (2024). MA. http://www.posit.co/

[CR50] Psychometric Theory (n.d.). *Google Books*. https://books.google.com/books/about/Psychometric_Theory.html?id=r0fuAAAAMAAJ. Accessed 5 July 2024.

[CR51] Pullmann, M. D., VanHooser, S., Hoffman, C., & Heflinger, C. A. (2010). Barriers to and supports of family participation in a rural system of care for children with serious emotional problems. *Community Mental Health Journal*, *46*(3), 211–220. 10.1007/s10597-009-9208-5 19551506 10.1007/s10597-009-9208-5 PMC2954890

[CR52] Raven, D., Jörg, F., Visser, E., Oldehinkel, A. J., & Schoevers, R. A. (2017). Time-to-treatment of mental disorders in a community sample of Dutch adolescents. A TRAILS study. *Epidemiology and Psychiatric Sciences*, *26*(2), 177–188. 10.1017/S2045796016000226 27075651 10.1017/S2045796016000226 PMC6998684

[CR53] Revelle, W. (2024). Procedures for Psychological, Psychometric, and Personality Research [R package psych version 2.4.6.26]. https://CRAN.R-project.org/package=psych. Accessed 5 July 2024.

[CR54] Rickwood, D. J., Mazzer, K. R., & Telford, N. R. (2015). Social influences on seeking help from mental health services, in-person and online, during adolescence and young adulthood. *BMC Psychiatry*, *15*, 40. 10.1186/s12888-015-0429-6 25886609 10.1186/s12888-015-0429-6 PMC4355380

[CR55] Rizopoulos, D. (2007). Ltm: An R package for latent variable modeling and item response analysis. *Journal of Statistical Software*, *17*, 1–25. 10.18637/jss.v017.i05

[CR56] Rosseel, Y. (2012). Lavaan: An R package for structural equation modeling. *Journal of Statistical Software,**48*(2), 1–36. 10.18637/jss.v048.i02

[CR57] Saraceno, B., van Ommeren, M., Batniji, R., Cohen, A., Gureje, O., Mahoney, J., et al. (2007). Barriers to improvement of mental health services in low-income and middle-income countries. *Lancet*, *370*(9593), 1164–1174. 10.1016/S0140-6736(07)61263-X 17804061 10.1016/S0140-6736(07)61263-X

[CR58] Sawyer, M. G., Rey, J. M., Arney, F. M., Whitham, J. N., Clark, J. J., & Baghurst, P. A. (2004). Use of health and school-based services in Australia by young people with attention-deficit/hyperactivity disorder. *Journal of the American Academy of Child and Adolescent Psychiatry*, *43*(11), 1355–1363. 10.1097/01.chi.0000138354.90981.5b 15502594 10.1097/01.chi.0000138354.90981.5b

[CR59] Sayal, K., Mills, J., White, K., Merrell, C., & Tymms, P. (2015). Predictors of and barriers to service use for children at risk of ADHD: Longitudinal study. *European Child & Adolescent Psychiatry*, *24*(5), 545–552. 10.1007/s00787-014-0606-z 25201055 10.1007/s00787-014-0606-z

[CR60] Schlack, R., Peerenboom, N., Neuperdt, L., Junker, S., & Beyer, A. K. (2021). The effects of mental health problems in childhood and adolescence in young adults: Results of the KiGGS cohort. *Journal of Health Monitoring*, *6*(4), 3–19. 10.25646/8863 35146318 10.25646/8863 PMC8734087

[CR61] Schmitt, T. A., Sass, D. A., Chappelle, W., & Thompson, W. (2018). Selecting the best factor structure and moving measurement validation forward: An illustration. *Journal of Personality Assessment,**100*(4), 345–362. 10.1080/00223891.2018.1449116 29630411 10.1080/00223891.2018.1449116

[CR62] Shivram, R., Bankart, J., Meltzer, H., Ford, T., Vostanis, P., & Goodman, R. (2009). Service utilization by children with conduct disorders: Findings from the 2004 Great Britain child mental health survey. *European Child & Adolescent Psychiatry,**18*(9), 555–563. 10.1007/s00787-009-0012-0 19353233 10.1007/s00787-009-0012-0

[CR63] Signorini, G., Singh, S. P., Boricevic-Marsanic, V., Dieleman, G., Dodig-Ćurković, K., Franic, T., et al. (2017). Architecture and functioning of child and adolescent mental health services: A 28-country survey in Europe. *The Lancet Psychiatry*, *4*(9), 715–724. 10.1016/S2215-0366(17)30127-X 28596067 10.1016/S2215-0366(17)30127-X

[CR64] Skrobinska, L., Newman-Taylor, K., & Carnelley, K. (2024). Psychosis and help-seeking behaviour-a systematic review of the literature. *Psychology and Psychotherapy,**97*(4), 583–605. 10.1111/papt.12531 39007652 10.1111/papt.12531

[CR65] Solmi, M., Radua, J., Olivola, M., Croce, E., Soardo, L., de Pablo, S., et al. (2022). Age at onset of mental disorders worldwide: Large-scale meta-analysis of 192 epidemiological studies. *Molecular Psychiatry,**27*(1), 281–295. 10.1038/s41380-021-01161-7 34079068 10.1038/s41380-021-01161-7 PMC8960395

[CR66] Stein, S. M., Christie, D., Shah, R., Dabney, J., & Wolpert, M. (2003). Attitudes to and knowledge of CAMHS: Differences between Pakistani and white British mothers: Original article: Attitudes to and knowledge of CAMHS. *Child and Adolescent Mental Health*, *8*(1), 29–33. 10.1111/1475-3588.00042 32797549 10.1111/1475-3588.00042

[CR67] Stylianidis, S., & Souliotis, K. (2019). The impact of the long-lasting socioeconomic crisis in Greece. *BJPsych International*, *16*(1), 16–18. 10.1192/bji.2017.31 30747163 10.1192/bji.2017.31 PMC6357520

[CR68] Teagle, S. E. (2002). Parental problem recognition and child mental health service use. *Mental Health Services Research*, *4*(4), 257–266. 10.1023/a:1020981019342 12558014 10.1023/a:1020981019342

[CR69] Terrence, D., Jorgensen, Pornprasertmanit, S., Alexander, M., Schoemann, & Rosseel, Y. (2022). *semTools: Useful tools for structural equation modeling*. https://CRAN.R-project.org/package=semTools

[CR70] Thornicroft, G., Sunkel, C., Alikhon Aliev, A., Baker, S., Brohan, E., El Chammay, R., et al. (2022). The lancet commission on ending stigma and discrimination in mental health. *The Lancet*, *400*(10361), 1438–1480. 10.1016/S0140-6736(22)01470-2 10.1016/S0140-6736(22)01470-2 36223799

[CR71] Thurston, I. B., & Phares, V. (2008). Mental health service utilization among African American and Caucasian mothers and fathers. *Journal of Consulting and Clinical Psychology*, *76*(6), 1058–1067. 10.1037/a0014007 19045973 10.1037/a0014007

[CR72] Topkaya, N., Şahin, E., & Meydan, B. (2016). The development, validity, and reliability of the barriers to seeking psychological help scale for college students. *International Journal of Sustainability in Higher Education*, *6*(1), 48. 10.5430/ijhe.v6n1p48

[CR73] Tzouvara, V., Papadopoulos, C., & Randhawa, G. (2016). Systematic review of the prevalence of mental illness stigma within the Greek culture. *The International Journal of Social Psychiatry*, *62*(3), 292–305. 10.1177/0020764016629699 26888966 10.1177/0020764016629699

[CR74] United Nations Publications (2014). *Children of the Recession: The Impact of the Economic Crisis on Child Well-being in Rich Countries*. UN. https://books.google.com/books/about/Children_of_the_Recession.html?hl=&id=jJLlrQEACAAJ

[CR75] Waller, G., Newbury-Birch, D., Simpson, D., Armstrong, E., James, B., Chapman, L., et al. (2023). The barriers and facilitators to the reporting and recording of self-harm in young people aged 18 and under: A systematic review. *BMC Public Health*, *23*(1), 158. 10.1186/s12889-023-15046-7 36694149 10.1186/s12889-023-15046-7 PMC9871435

[CR76] Whitney, D. G., & Peterson, M. D. (2019). US national and state-level prevalence of mental health disorders and disparities of mental health care use in children. *JAMA Pediatrics,**173*(4), 389–391. 10.1001/jamapediatrics.2018.5399 30742204 10.1001/jamapediatrics.2018.5399 PMC6450272

[CR77] World Health Organization (2021). *Mental health atlas 2020. Geneva*.

[CR78] World Health Organisation (2020). *Covid-19 disrupting mental health services in most countries who survey*.

[CR79] Xiong, T., Kaltenbach, E., Yakovenko, I., Lebsack, J., & McGrath, P. J. (2022). How to measure barriers in accessing mental healthcare? Psychometric evaluation of a screening tool in parents of children with intellectual and developmental disabilities. *BMC Health Services Research*, *22*(1), 1383. 10.1186/s12913-022-08762-0 36411458 10.1186/s12913-022-08762-0 PMC9677628

[CR80] Yurgelun-Todd, D. (2023). How parents can help or hinder access to mental health services for young people. *Children and Youth Services Review*, *145*, 106760. 10.1016/j.childyouth.2022.106760

[CR81] Zanus, C., Battistutta, S., Aliverti, R., Montico, M., Cremaschi, S., Ronfani, L., et al. (2017). Adolescent admissions to emergency departments for self-injurious thoughts and behaviors. *PLoS One,**12*(1), e0170979. 10.1371/journal.pone.0170979 28125701 10.1371/journal.pone.0170979 PMC5268645

