# Supplementary material for: Barriers to Access to Care Evaluation Scale - Proxy Report (BACE-PR): Evidence of Reliability and Validity for Caregivers Reporting on Children and Adolescents with Mental Health Concerns in Greece
Source: Adm Policy Ment Health. 2025 Aug 25;52(5):983–98. doi: 10.1007/s10488-025-01466-7 (PMC12449398; doi:10.1007/s10488-025-01466-7)
Supplement: Supplementary file 1 — Supplementary Material 1 [file 10488_2025_1466_MOESM1_ESM.pdf]

## Supplemental Material

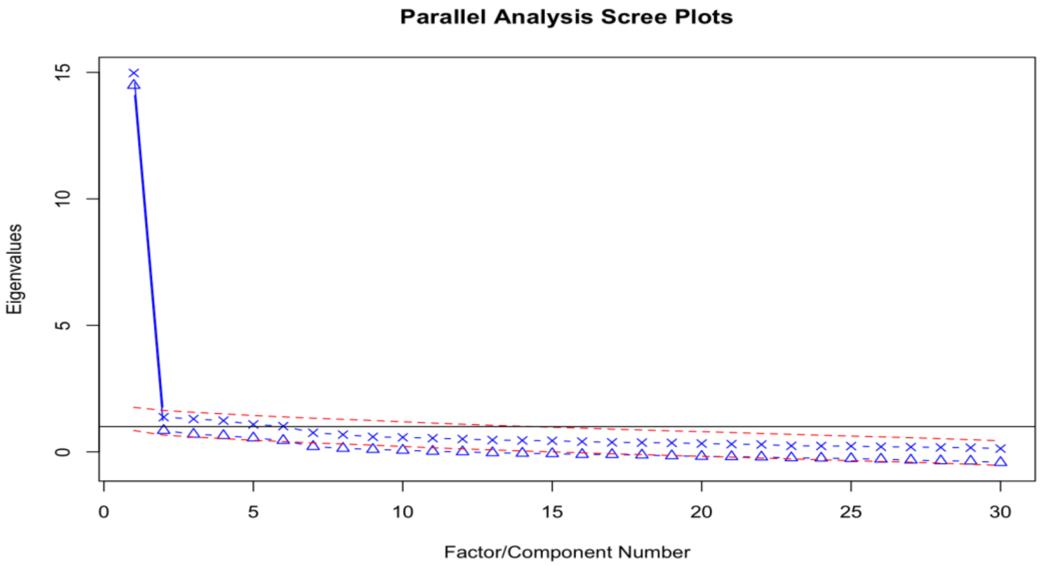


**Supplemental Fig. S1** Parallel analysis plot

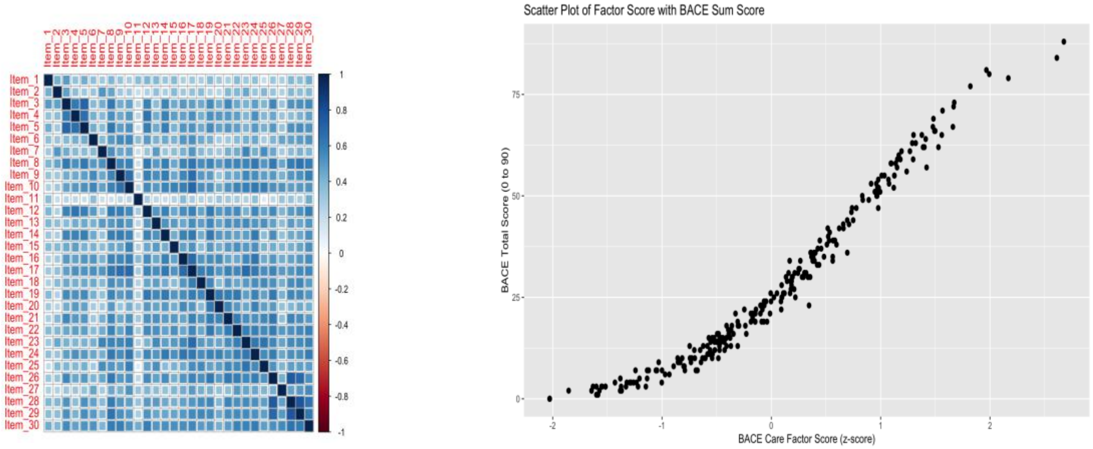


**Supplemental Fig. S2** Correlation matrix and scatter plot showing the association between summed score and IRT-based score

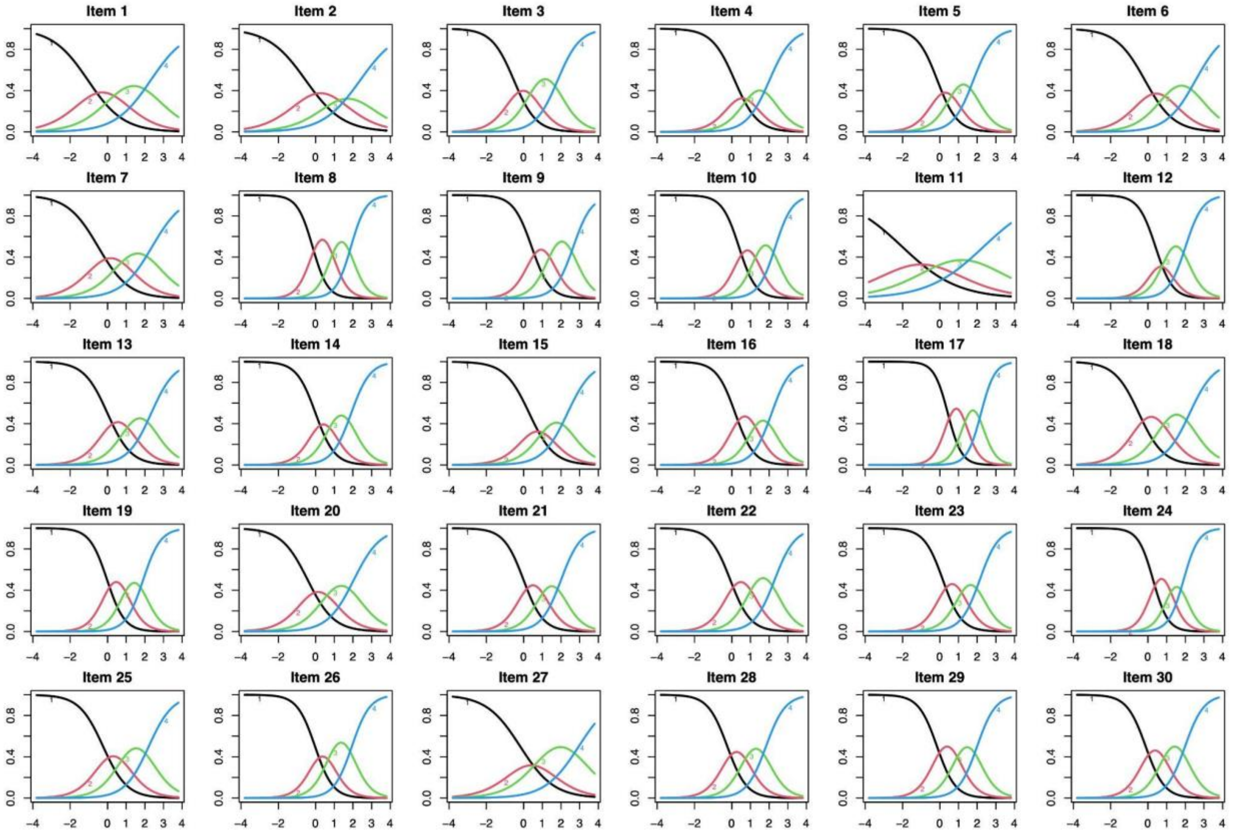


**Supplemental Fig. S3** Item Response Characteristic Curves (unidimensional solution)

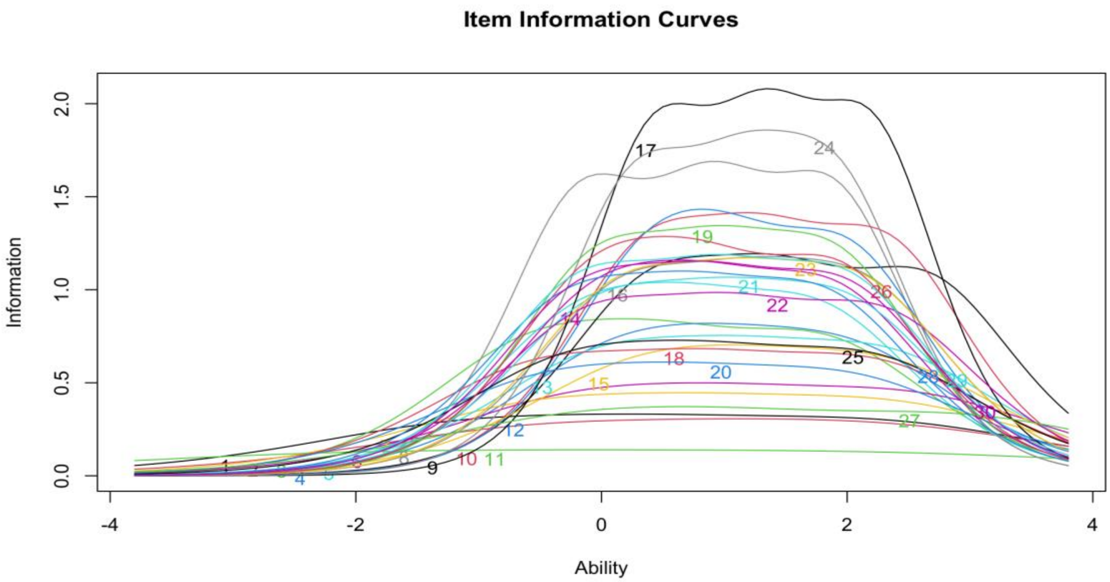


**Supplemental Fig. S4** Item Information Curves (unidimensional solution)

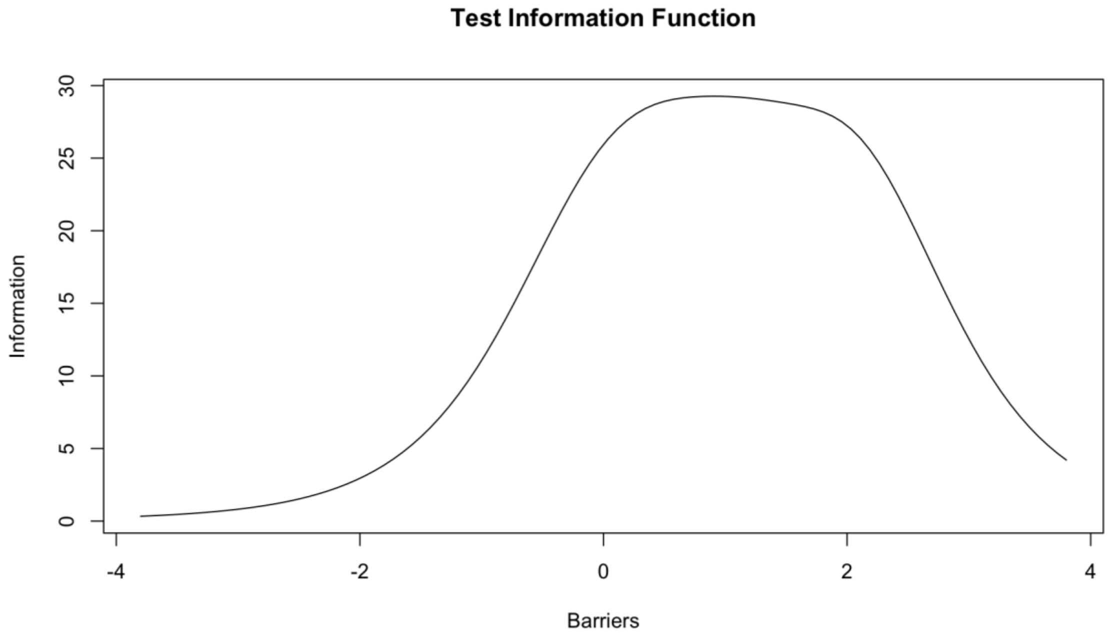


**Supplemental Fig. S5** Test Information Function of the Barriers to Access to Care Evaluation (unidimensional solution)
